# Multiple whitish papules on the posterior neck of an elderly woman^☆^^☆☆^

**DOI:** 10.1016/j.abd.2019.05.003

**Published:** 2019-12-18

**Authors:** Joana Calvão, Bárbara Roque Ferreira, José Carlos Cardoso

**Keywords:** Elastic tissue, Neck, Pseudoxanthoma elasticum

## Abstract

White fibrous papulosis of the neck is a rare entity, with fewer than 50 cases described. It is a benign pathology whose main interest lies in its broad differential diagnosis, especially with pseudoxanthoma elasticum. The authors report the case of a 77-year-old woman with multiple yellow-white monomorphic papules on the posterior cervical region, with years of evolution. Cutaneous biopsy revealed a nodular area in the superficial and middle reticular dermis, with slight thickening of the collagen fibers and focally enlarged elastic fibers, aspects highlighted in the Verhoeff staining that additionally showed absence of elastic fibers in the papillary dermis.

## Case report

**Figure 1:**
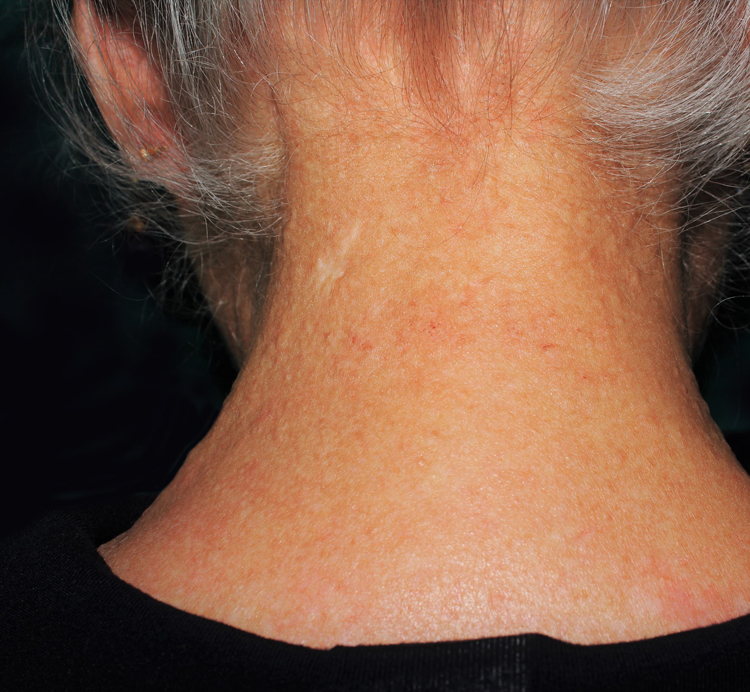
Multiple small whitish papules on the posterior cervical region.

**Figure 2:**
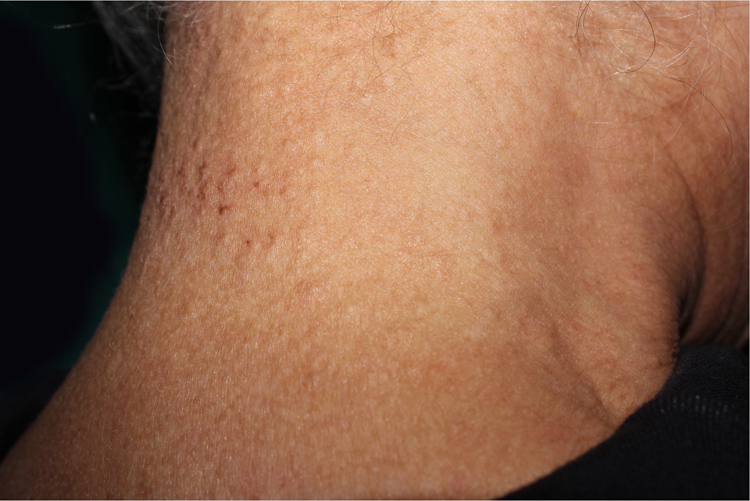
Multiple small whitish papules on the posterior cervical region.

**Figure 3:**
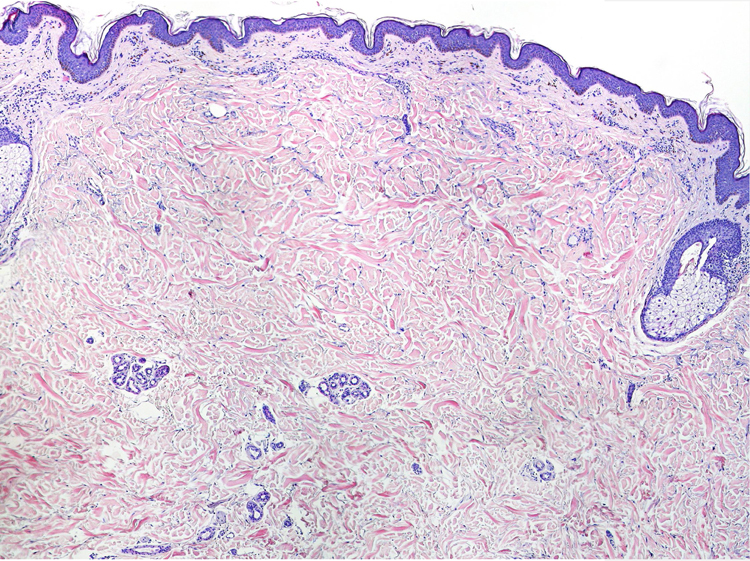
Mild solar elastosis and thickening of collagen bundles in the superficial and mid-reticular dermis (Hematoxylin & eosin, ×40).

**Figure 4:**
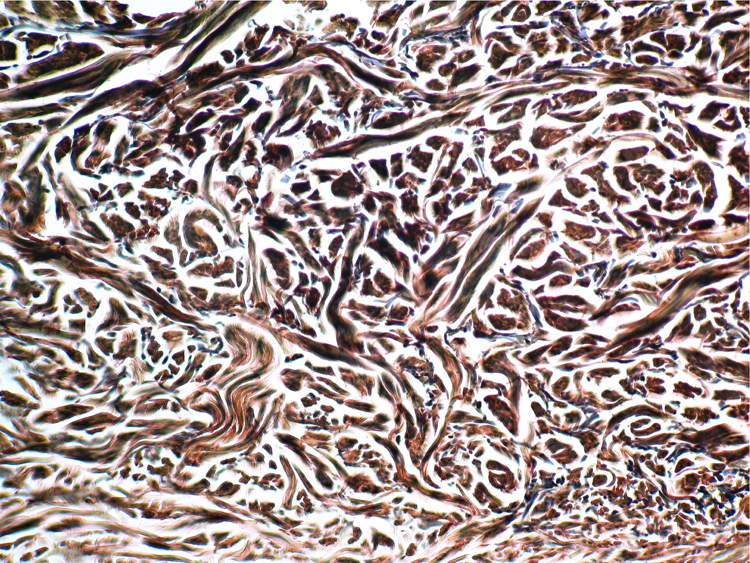
Thickening of collagen bundles and multifocal coarse thickening of the elastic fibers (Verhoeff, ×200).

A 77-year-old woman, who was otherwise healthy, presented with multiple white-yellowish millimetric, monomorphic, non-follicular papules, on the posterior cervical region (Figure 1, Figure 2). These lesions had been present for several years, and except for mild pruritus, were asymptomatic. Physical examination, including peripheral pulses and cardiovascular evaluation, revealed normal findings. Cutaneous biopsy revealed an ill-defined nodular area in the superficial and mid-reticular dermis, characterized by slight thickening of the collagen fibers and by focally enlarged elastic fibers (Fig. 3). These findings were highlighted in the Verhoeff-Van Gieson staining, which further showed absence of elastic fibers in the papillary dermis (Fig. 4). Calcification of elastic tissue was not seen.

## Discussion

Clinicopathological correlation favored the hypothesis of white fibrous papulosis of the neck (WFPN). In this context, the authors decided on a watch-and-wait approach; the lesions currently remain stable.

WFPN was first described by Shimizu et al. in 1985. A few years later, the same authors reported this condition in a group of 32 Japanese patients.^1^ Although initially reported mainly in Japanese men, it also affects Caucasian women, from the 5th to the 9th decades of life.^2^ WFPN is a rare acquired fibroelastolytic disorder, which presents clinically as yellowish-white round or oval papules that occur more frequently on the posterior neck region, but also on the back, without associated systemic manifestations. Its etiopathogenesis, not fully understood, seems to be related to intrinsic skin photoaging, but is probably multifactorial.^3^, ^4^ The clinical relevance of this entity lies in its broad differential diagnosis, especially with pseudoxanthoma elasticum (PXE), a genetic disease caused by a mutation in the ABCC6 (ATP-binding cassette sub-family C member 6) gene and is associated with ectopic mineralization of the skin, eyes, and blood vessels.^5^, ^6^ However, in contrast to the latter, WFPN usually appears late in life, is not associated with systemic complications, and does not require further investigation.^3^ Histological examination reveals dermal fibrosis consisting of circumscribed areas of thickened collagen bundles in the papillary and mid-reticular dermis, as well as a variable loss of dermal elastic tissue.^3^, ^6^ Despite its benign nature, WFPN may be cosmetically undesirable and sometimes itchy. Until now, no effective treatments have been established.^2^ Some topical agents like tretinoin and antioxidants were tried, but improvement was not notable.^2^, ^7^ Surgical excision may be an alternative choice of treatment in cases with circumscribed lesions.^2^ Moreover, since WFPN is arguably a feature of photoaging or intrinsic aging, fractional non-ablative laser has been recently tried with good results.^2^ However, none of the treatments present sufficient evidence in the literature.

## Financial support

None declared.

## Authors’ contribution

Joana Calvão: Approval of the final version of the manuscript; composition of the manuscript; collection, analysis, and interpretation of data; participation in the design of the study; intellectual participation in the propaedeutic and/or therapeutic conduct of the studied cases; critical review of the literature; critical review of the manuscript.

Bárbara Roque Ferreira: Approval of the final version of the manuscript; collection, analysis, and interpretation of data; participation in the design of the study; critical review of the manuscript.

José Carlos Cardoso: Approval of the final version of the manuscript; conception and planning of the study; collection, analysis, and interpretation of data; critical review of the manuscript.

## Conflicts of interest

None declared.

## References

[1] Shimizu H., Kimura S., Harada T., Nishikawa T. (1989). White fibrous papulosis of the neck: a new clinicopathologic entity?. J Am Acad Dermatol.

[2] Lueangarun S., Panchaprateep R. (2016). White fibrous papulosis of the neck treated with fractionated 1550-nm erbium glass laser: a case report. J Lasers Med Sci.

[3] Cunha N., Cabete J., João A., Lencastre A. (2018). The spectrum of fibroelastolytic papulosis: a retrospective case series. Revista SPDV.

[4] Rita T., Oliveira M. (2017). Pseudoxanthoma elasticum-like papillary dermal elastosys. An Bras Dermatol.

[5] Germain D.P. (2017). Pseudoxanthoma elasticum. Orphanet J Rare Dis.

[6] Hosen M.J., Lamoen A., De Paepe A., Vanakker O.M. (2012). Histopathology of pseudoxanthoma elasticum and related disorders: histological hallmarks and diagnostic clues. Scientifica (Cairo).

[7] Gencoglan G., Ceylan C., Kazandi A.C. (2011). White fibrous papulosis of the neck. Cutan Ocul Toxicol.

